# 'Bois noir' phytoplasma induces significant reprogramming of the leaf transcriptome in the field grown grapevine

**DOI:** 10.1186/1471-2164-10-460

**Published:** 2009-10-02

**Authors:** Matjaž Hren, Petra Nikolić, Ana Rotter, Andrej Blejec, Nancy Terrier, Maja Ravnikar, Marina Dermastia, Kristina Gruden

**Affiliations:** 1 National Institute of Biology, Department of Biotechnology and Systems Biology, Večna pot 111, 1000 Ljubljana, Slovenia; 2 UMR SPO, Campus Agro-M/INRA, 2 Place Viala, 34060 Montpellier, Cedex 01, France

## Abstract

**Background:**

Phytoplasmas are bacteria without cell walls from the class *Mollicutes*. They are obligate intracellular plant pathogens which cause diseases in hundreds of economically important plants including the grapevine (*Vitis vinifera*). Knowledge of their biology and the mechanisms of their interactions with hosts is largely unknown because they are uncultivable and experimentally inaccessible in their hosts. We detail here the global transcriptional profiling in grapevine responses to phytoplasmas. The gene expression patterns were followed in leaf midribs of grapevine cv. 'Chardonnay' naturally infected with a phytoplasma from the stolbur group 16SrXII-A, which is associated with the grapevine yellows disease 'Bois noir'.

**Results:**

We established an on field experimental system in a productive vineyard that allowed application of molecular tools in a plant natural environment. Global transcription profiles of infected samples were compared with the healthy ones using microarray datasets and metabolic pathway analysis software (MapMan). The two-year-long experiment revealed that plant genes involved in primary and secondary metabolic pathways were changed in response to infection and that these changes might support phytoplasma nutrition. A hypothesis that phytoplasmas interact with the plant carbohydrate metabolism was proven and some possibilities how the products of this pathway might be utilized by phytoplasmas are discussed. In addition, several photosynthetic genes were largely down-regulated in infected plants, whereas defense genes from the metabolic pathway leading to formation of flavonoids and some PR proteins were significantly induced. Few other genes involved in defense-signaling were differentially expressed in healthy and infected plants. A set of 17 selected genes from several differentially expressed pathways was additionally analyzed with quantitative real-time PCR and confirmed to be suitable for a reliable classification of infected plants and for the characterization of susceptibility features in the field conditions.

**Conclusion:**

This study revealed some fundamental aspects of grapevine interactions with the stolbur 'Bois noir' phytoplasma in particular and some plant interactions with phytoplasmas in general. In addition, the results of the study will likely have an impact on grape improvement by yielding marker genes that can be used in new diagnostic assays for phytoplasmas or by identifying candidate genes that contribute to the improved properties of grape.

## Background

Phytoplasmas are plant pathogenic bacteria belonging to the class *Mollicutes *[1]. They are cell wall-free, and both their cell size (0.1-0.8 μm in diameter) and genome size (0.5-1.3 Mbp) are the smallest among bacteria. They are transmitted from plant to plant by sap-feeding insect vectors and they propagate within the cytoplasm of both insects and plants. In plants they exclusively inhabit nutrient-rich phloem tissues, where they have been documented by electron microscopy in sieve elements, companion cells and phloem parenchyma cells [2]. Phytoplasmas have a broad range of plant hosts among monocots and dicots and diseases of many important crops have already been associated with these pathogens [1]. Since the discovery of phytoplasmas four decades ago, attempts to culture them in cell-free media have failed, making it difficult to determine the taxonomic status of phytoplasmas by the traditional methods applied to cultured prokaryotes. Although satisfaction of Koch postulates has not yet been achieved, in order to start formal classification of phytoplasmas the '*Candidatus *Phytoplasma' genus has been proposed and adopted [3]. However, some phytoplasmas have had an informal description, but they have not been validly described. For them the practice of naming a phytoplasma on the basis of the associated plant syndrome is still valid [3].

Recent progress in the sequencing of four phytoplasma genomes and their comparative analysis [4-7] have increased our understanding of phytoplasma genetics and revealed that these plant pathogens experienced substantial evolutionary gene decay, gene loss, and disruption or loss of important biosynthetic pathways [8]. These genomic changes may account for their complete reliance on the host plant or insect cells for survival.

As no phytoplasma cultures are available, not all the approaches can be applied and studies on phytoplasma interactions with host plants are limited. However, the latter have been mostly studied in periwinkle (*Cataranthus roseus*). Phytoplasmas can be transmitted to periwinkle via graft inoculation or via their respective insect vectors [9]. Periwinkle is the first choice also because phytoplasmas concentration can reach very high level and infected plants are easy to propagate. Considering the extreme difficulties associated with the establishment of an accurate and reliable experimental system, very little is also known about the mechanisms of phytoplasma phytopathogenicity [1,10]. This is especially true for phytoplasmal diseases of major crops, including woody grapevine (*Vitis vinifera *L.), which is, on a global basis, both the most widely cultivated and economically important fruit crop.

In grapevine, phytoplasma cause grapevine yellows (GY) diseases, which were identified in the majority of grapevine growing countries worldwide. Several molecularly distinct phytoplasma groups which cause the GY were identified; however, a phytoplasma from the stolbur group/16SrXII-A [3], associated with the GY disease 'Bois noir', is very common. The name for this phytoplasma '*Ca*. P. solani' has been proposed at the X International Congress of the International Organization of Mycoplasmology (1994), but the phytoplasma has not been formally described yet. Accordingly it is referred to as 'Bois noir' ('BN') phytoplasma. Typical symptoms of all phytoplasma infection in grapevine are leaf curling and discoloration of leaf veins and laminas, interveinal yellowing or reddening, according to the variety, uneven or total lack of lignification of canes, flower abortion and berry withering. Leaves on the affected shoots often have a hard, brittle texture.

In order to demonstrate the potential of grapevine plants infected with 'BN' phytoplasma to interact with their pathogen, we present here the qualitative and quantitative changes in the global gene expression profiles of the healthy and infected plants of cv. 'Chardonnay'. The experiment was carried out in a production vineyard over two successive growing seasons. Whereas studies of plant responses to pathogen infection conducted under controlled laboratory or glasshouse conditions can focus on the impact of individual factors, it is the resulting changes in the transcript profile of the field established system that potentially uncovers the complexity of signaling networks involved in the pathogenicity [11]. A complementary experimental system for analyzing gene expression profiling in the field grown 'BN' phytoplasma infected grapevine has been described very recently [12]. The present work is an extension of our recent study on three genes encoding sucrose synthase, alcohol dehydrogenase 1 and Hsp70 that are differentially expressed in different grapevine cultivars infected with phytoplasmas [13], to a high throughput transcriptome analysis of the infected cv. 'Chardonnay'. We analyzed the traits of infection and further evaluated the potential contribution of several metabolic/signaling host pathways to the infection status. Based on the assumption that phytoplasmas mobilize phosphorylated hexoses from the companion cells of phloem [2] and according to accumulating evidence that levels of reducing sugars and sucrose are generally higher in the source leaves of plants, which are infected with different phytoplasma species [14-18], our focus was on the gene expression level of plant enzymes involved in carbohydrate metabolism and photosynthesis. Presumably its changes in infected plants are related to the requirements of phytoplasma for energy and growth. Our collective findings contribute towards understanding the potential main steps in phytoplasma pathogenicity pathway.

## Results

### Correlation between phytoplasma presence in leaf mid-rib and expression of disease symptoms

To investigate the distribution and persistence of 'BN' phytoplasma in naturally infected grapevines of cv. 'Chardonnay' grown in a production vineyard, the same plants were sampled in two subsequent growing seasons. In the first growing season the plants were randomly chosen for sampling from the vineyard plants to eliminate potential bias caused by environmental factors other than phytoplasma infection. However, among those plants only those with apparent phenotype of the 'BN' disease (stage 4 on the scale of 0 to 4) [see Additional file 1] and clear leaf symptoms of 'BN' phytoplasma infection [see Additional file 2] were chosen for the group referred to as infected, and plants with asymptomatic leaves for the healthy group. By using reverse transcription coupled quantitative real-time PCR (qRT-PCR), 'BN' phytoplasma was detected in all but one sample classified as infected, and all asymptomatic samples were phytoplasma free [see Additional file 1]. In the following season the same plants were sampled. In general the symptom expression was very mild and at most phenotype of stage 2 occurred. In the second season seven samples from the healthy group in the first season had exhibited phenotype of stage 1. In none of them 'BN' phytoplasma was detected by the qRT-PCR. However, in two of them GFkV was detected by ELISA and in the third one GLRaV was present in addition to GFkV [see Additional file 1]. In additional 10 samples with phenotype 1 or 2, which were taken from plants infected with 'BN' phytoplasma in the previous season [see Additional file 1], phytoplasmas were not detected by the qRT-PCR. Six samples from different plants were phytoplasma-free in the second growing season (Plant.ID 17, 20, 23) [see Additional file 1]. Those plants might be in the process of recovery. Seven leaf samples collected in both seasons were from plants that had another shoot, which was 'BN' phytoplasma positive [see Additional file 1]. This observation supports the study on 'BN' phytoplasma distribution in different grapevine cultivars, which shows that very low proportion of asymptomatic leaf samples on otherwise infected plants are 'BN' phytoplasma positive [19]. This may indicate an uneven distribution of phytoplasmas inside the plant and their sporadic systemic spreading throughout grapevines [19] and thus a more indirect influence on the host metabolism. Such indirect influence has been proposed in papayas infected with stolbur '*Ca*. P. australiense' causing Australian papaya dieback [15]. In the second growing season only samples where phytopasmas were detected by the qRT-PCR, were considered as infected ones.

### Microarray analysis and functional categorization of differentially expressed genes in phytoplasma infected grapevines

A complex microarray data analysis of samples collected only in the first growing season was applied to determine global transcriptional changes occurring in grapevine leaf tissues after the natural infection with 'BN' phytoplasma. From approximately 14,500 grapevine genes, for which expression could be determined with microarrays used in our experiments, 2,456 genes were found to be differentially expressed (DE) [see Additional file 3] in the infected plants: 54% genes were up-regulated (fold change range: 1.2 to 45.0) and 46% of genes were down-regulated (fold change range: -1.2 to -26.0). The final list of DE genes obtained in microarray data analysis was imported into a plant-specific MapMan gene ontology, which enables simpler and visually enhanced analysis by biological processes and supports an ontology-based statistical data analysis [20]. Therefore, it facilitates biological interpretation and provides users with a global overview of the results. DE genes were mapped into 32 out of 34 pathways/processes represented in MapMan and the most significantly altered are shown in Table 1. However, 1,129 DE genes (46%) did not have a reliable annotation and were not assigned to any pathway/process.

**Table 1:** Significantly altered processes or protein families according to the changes in gene expression level in symptomatic compared to asymptomatic grapevine leaf samples.

**Upregulated processes/protein families**	**Number of genes**	**p-value**	**Downregulated processes/protein families**	**Number of genes**	**p-value**
Cell organization	24	4.0 × 10^-4^	Photosynthesis	97	< 10^-20^
	
Cell wall	55	1.0 × 10^-4^	TCA/org. transformation-carbonic anhydrases	4	4.6 × 10^-3^
	
β-1,3 glucan hydrolases	10	1.1 × 10^-2^	N-metabolism	6	3.5 × 10^-2^
			
Biotic stress	41	4.2 × 10^-3^	N-metabolism - ammonia metabolism - glutamate synthase	3	8.4 × 10^-3^
			
Secondary metabolism - flavonoids	15	1.2 × 10^-3^	Secondary metabolism - isoprenoids terpenoids	3	5.3 × 10^-3^
			
Secondary metabolism - chalcones	4	1.3 × 10^-2^	Protein assembly and cofactor ligation	5	9 × 10^-3^
			
Amino acid metabolism	47	5 × 10^-2^	Protein-synthesis - plastid protein	5	3.1 × 10^-2^
Amino acid metabolism -degradation - aspartate family - methionine	5	7.7 × 10^-3^	Protein-folding	12	3.8 × 10^-2^
			
Amino acid metabolism - synthesis	23	1.9 × 10^-2^	GDSL-motif lipase	8	2.9 × 10^-2^
			
Protein degradation - subtilases	3	2.0 × 10^-2^	Transport-ABC transporters and multidrug resistance systems	9	3.6 × 10^-2^
	
Minor charbohydrate metabolism	22	1.4 × 10^-2^	DNA unspecified	4	3.2 × 10^-2^
			
Major charbohydrate metabolism - degradation - sucrose	5	1.6 × 10^-2^	RNA - regulation of transcription.C2C2(Zn) CO-like, Constans-like zinc finger family	8	2.3 × 10^-2^
			
RNA - regulation of transcription - GRAS transcription factor family	4	3.0 × 10^-2^	Regulation of transcription - MYB-related transcription factor family	11	5.8 × 10^-5^
Development	41	3.1 × 10^-2^	Regulation of transcription - Aux/IAA family	12	1.3 × 10^-3^
Glycolysis	20	9.8 × 10^-4^			

The results are part of the MapMan gene ontology showing numbers of genes annotated to each process or family (indent) and corresponding p-values (as calculated by MapMan Wilcoxon rank sum test).

### Detailed analysis of in-field grown plants using qRT-PCR

We performed reverse transcription coupled with qPCR assays on a subset of genes to verify the differential expression estimated with microarray analysis and to determine the degree of change in transcript abundance of those genes in individual samples in two subsequent seasons. To get a better insight into different host metabolic pathways, the following genes were selected from the list of genes from the microarray study that showed statistically significant differential expression between 'BN' phytoplasma infected and healthy samples: a gene involved in photosynthesis (*VvAcyt*, encoding apocytochrome f precursor), genes involved in sugar metabolism (*VvInv2 *encoding vacuolar acid invertase 2; *VvAgpL *encoding large subunit of ADP-glucose pyrophosphorylase; *VvSuSy *encoding sucrose synthase), alcohol dehydrogenase 1 being involved in NAD regeneration in hypoxic conditions (*VvAdh1*), genes with possible roles in signaling (*VvLox *encoding lipoxygenase; *VvEtr *encoding ethylene receptor; *VvHP *encoding histidine-containing phosphotransfer protein involved in cytokinin signal transduction; *VvSamt *encoding S-adenosyl-L-methionine: salicylic acid carboxyl methyltransferase), a gene involved in secondary metabolism (*VvF3h *encoding flavanone 3-hydroxylase), a gene involved in regulation of transcription (*VvWrky *encoding transcription factor WRKY54,), one of the pathogenesis-related proteins (PR-proteins) genes found to be differentially expressed in our experimental study (*VvOlp *encoding osmotin protein), a gene involved in the callose formation (*VvCaSy *encoding callose synthase), a gene involved in cytokinin degradation (*VvCko *encoding cytokinin oxidase) and three genes encoding three different β-1,3-glucanases, which hydrolyze β-1,3-glucans (*VvGlc1*, *VvGlc2 *and *VvGlc3*). The expression of *VvSuSy *and *VvAdh1*in the first season have been reported previously [13] and were here re-examined to show the comparability of analyses and expanded to the following season.

**Figure 1:**
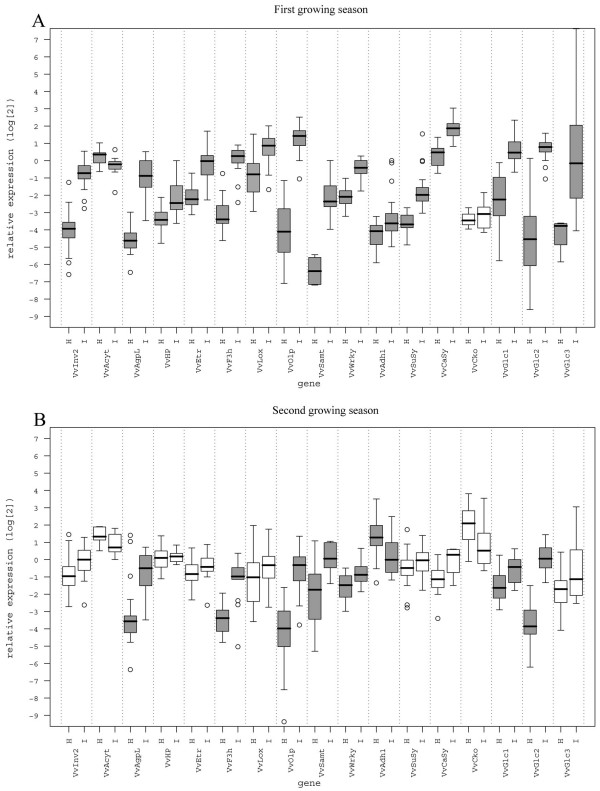
**Boxplots showing relative expression ratios**. Boxplots showing relative expression ratios (log_2 _transformed) of genes *VvvInv2*, *VvAcyt*, *VvAgpL*, *VvHP*, *VvEtr*, *VvF3h*, *VvLox*, *VvOlp*, *VvSamt*, *VvWrky*, *VvAdh1*, *VvSuSy*, *VvCaSy*, *VvCko*, *VvGlc1*, *VvGlc2 *and *VvGlc3 *normalized using 18S/Cox (geometric mean of 18S and Cox Ct values) in healthy and phytoplasma-infected cv. 'Chardonnay' samples (leaf veins) collected in the first growing season (A) and in the second growing season (B). Threshold for statistical significance between relative expression ratios of healthy and infected samples is P < 0·05 (Welch two sample t-test). Boxplots for genes where such differences were observed are represented with dark gray colors in contrast to white boxplots for non-significant genes.

The results of the qRT-PCR analysis of samples which originated from plants grown in the first examined season were in agreement with those of microarray experiment [see Additional files 1, 3]. Most of the investigated genes except *VvCko *were significantly up-regulated in the group of infected samples relative to the healthy one. Only gene *VvAcyt *was significantly down-regulated (Figure 1A). It is worth noting that the levels of *VvSamt *and *VvInv2 *gene expression in the healthy group of the first growing season were below the limit of accurate quantification by qRT-PCR. However, transcripts were highly abundant in the infected leaf tissue. The most significant difference in gene expression was detected in the *VvOlp *gene (Figure 1A). In the second growing season the trend in gene expression was similar, and the differences in gene expression were significant for eight out of 17 genes, specifically for *VvAgpL*, *VvF3h*, *VvOlp*, *VvSamt, VvWrky, VvAdh1, VvGlc1 *and *VvGlc2*. However, the trend was inverted for the transcript of gene encoding alcohol dehydrogenase 1. In the second growing season it was significantly down-regulated (Figure 1B).

### Disease status classification of samples based on qRT-PCR gene expression results

**Figure 2:**
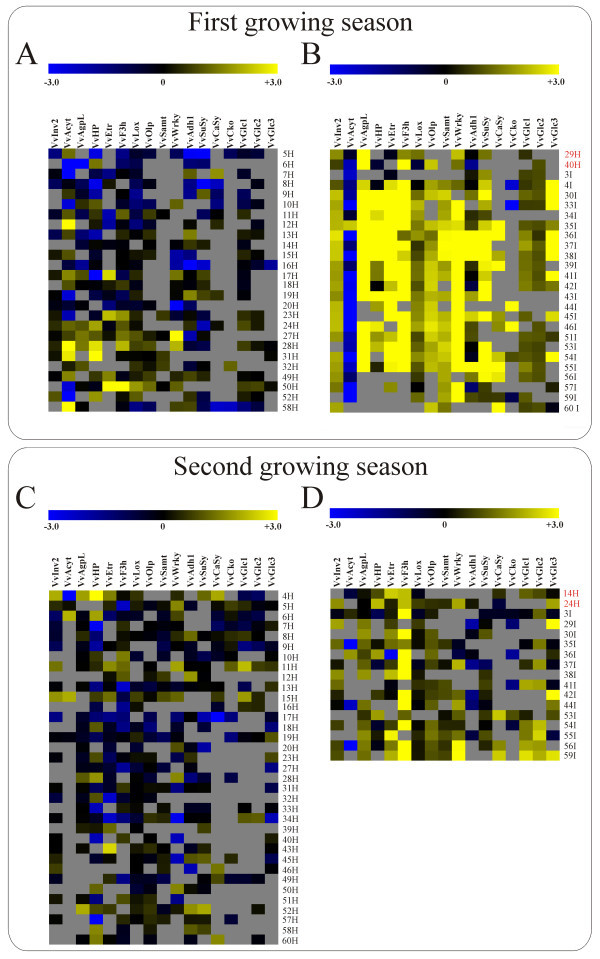
**Support vector machines**. Support vector machines classification of 52 samples from the first and the second growing season into two groups (healthy and infected samples) presented as heatmaps (A-D). Scaled relative expression values of each gene are coded by color (-3 blue, +3 yellow). Samples with sample-names in red were misclassified.

Samples were classified as either healthy or infected based on the qRT-PCR gene expression results by using the support vector machines algorithm (SVM) (Figures 2A, B). In both growing seasons only a few samples without detected phytoplasmas were classified as infected (i.e. Sample.ID 29, 40/1^st ^growing season; Sample.ID 14, 24/2^nd ^growing season) [see Additional file 1]. Samples with IDs 40 and 14 with expressed phenotypes 4 and 1, respectively, were infected with a virus GFkv. However, in grapevine GFkV is generally latent and asymptomatic, which has been also shown in the study of 'Chardonnay' plants infected with 'BN' phytoplasma [12]. Accordingly, its presence is not likely the reason for misclassification. On the other hand, the zero phenotype of the misclassified sample with ID 24 was not tested for viruses [see Additional file 1]. Sample.ID 29, with a phenotype zero was collected from an asymptomatic shoot on the infected plant (Plant.ID 15) [see Additional file 1]. The possible reason for sorting these samples among infected samples might be an undetectably low amount of phytoplasma or a latent virus infection. Nevertheless, most of the samples with or without 'BN' phytoplasma, which proved to be virus positive [see Additional file 1] were categorized correctly into the healthy or infected group. We concluded that in general infections with tested viruses do not affect gene expression of the selected genes. Accordingly, such samples were not excluded from the analysis.

Although it seems that the chosen set of reference genes is reliable for classification of samples infected with 'BN' phytoplasma, additional genes might improve its accuracy and robustness.

### Changes in genes involved in carbohydrate metabolism and glycolysis

Hexoses are produced from sucrose by vacuolar invertase and sucrose synthase. These enzymes are synthesized in source leaves and transported via the phloem to sink tissues. As shown by microarray analyses the transcripts of their genes were up-regulated upon 'BN' phytoplasma infection [see Additional file 3]. Specifically, a transcript level of *VvInv2 *encoding grapevine acid vacuolar invertase 2 (Vv_10004311), which hydrolyzes sucrose to glucose and fructose, increased by 4.9-fold; and that of *VvSusy *(Vv_10000177) encoding sucrose synthase, which catalyzes reversible formation of UDP-glucose and fructose from UDP and sucrose, increased by 2.5-fold. The microarray study also revealed a significant increase (by 2.8-fold) of the transcript of the gene for callose synthase (*VvCasy*, Vv_10001615), which is involved in the callose formation and the increase of three genes belonging to a cluster of β-1,3-glucanases (Vv_10000389, Vv_10010418, Vv_10002068), which hydrolyze β-1,3-glucans (4.3 to 11.0-fold increase). All these changes had been confirmed by qRT-PCR assay in both examined growing seasons (Figure 1).

We found that the expression level of genes encoding enzymes involved in the interconversions between the sugar phosphorylated forms increased in leaves with a phenotype 4 in comparison with the phenotype zero (i.e. phosphoglucomutase, Vv_10003926: 1.7- fold change; phosphoglucose isomerase, Vv_10011330/Vv_10000192: 2.0/1.9-fold change; pyrophosphate-dependent phosphofructokinase, Vv_10008686: 1.7-fold change; fructose 1.6-bisphosphate aldolase, Vv_10003742: 1.6-fold change; glyceraldehyde-3-phosphate dehydrogenase, Vv_10004364/Vv_10004365/Vv_10008871: 2.8/2.3/3.7-fold change; phosphoglycerate kinase, Vv_10003833: 1.7-fold change) [see Additional file 3].

Among the genes associated with starch synthesis, the expression of gene for a large subunit of ADP-glucose pyrophosphorylase (*VvAgpL*) was significantly induced in infected samples compared to the phytoplasma-free ones (Vv_10001368, 11.0-fold change) [see Additional file 3]. The transcript abundance of *VvAgpL *was also proven with qRT-PCR in two growing seasons (Figure 1).

### Photosynthesis is affected in infected grapevines

**Figure 3:**
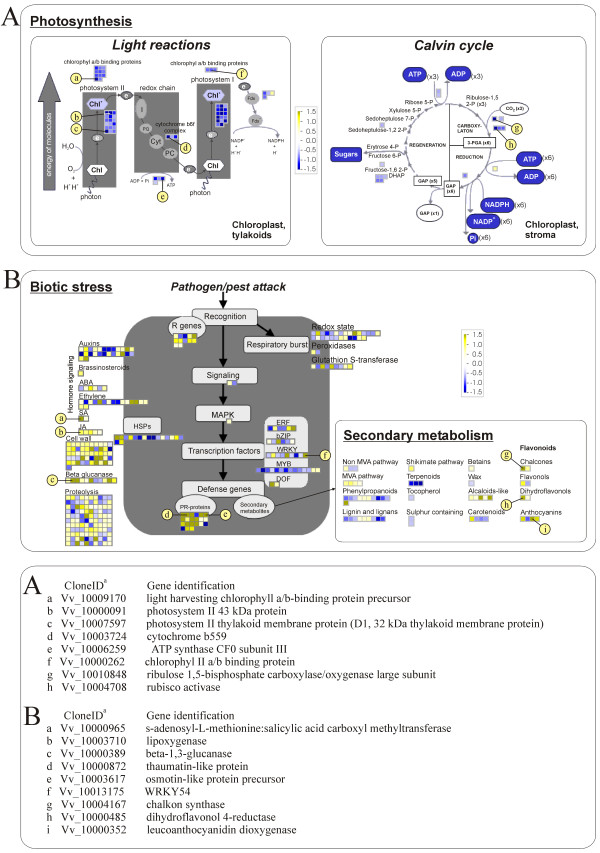
**A schematic representations of genes involved in photosynthesis**. (A), biotic stress and secondary metabolism (B). Each gene is represented by a colored square. Yellow color represents up-regulation and blue color down-regulation in infected vs. healthy samples. A few most interesting genes are pointed out. Schemes are slightly adapted visualizations of MapMan pathways. Only differentially expressed genes are presented.

Several genes involved in light reactions of photosynthesis were significantly down-regulated in infected plants (Figure 3). 29 of these genes were associated with photosystem II, three of them encoded proteins of cytochrome b6/f complex, four ATP synthase and 20 genes were part of the photosystem I. In addition, the genes for the large subunit of Ribulose 1.5-bisphosphate carboxylase/oxygenase (*VvrRbcL*) and Rubisco activase (*VvRca*) in the Calvin cycle were also significantly affected by the infection (Figure 3) [see Additional file 3].

### Defense and/or pathogenesis related genes change upon 'BN' phytoplasma infection

In infected leaves, 21 genes encoding pathogenesis-related proteins of several classes were induced (Figure 3), [see Additional file 3]. Among them, genes encoding osmotin and thaumatin-like proteins from the family of pathogenesis-related proteins of class 5 (PR-5) and several chitinase isoenzymes were significantly up-regulated in comparison with healthy samples, as shown by microarray analysis (Figure 3), [see Additional file 3]. We specifically showed a relevance of these results through the qRT-PCR analysis of osmotin gene expression (*VvOlp*, PR-5 class) in both growing seasons (Figure 1).

Genes for major enzymes in the flavonoid biosynthetic pathway (i.e. chalcone synthase, *VvCHS*, Vv_10004167; flavanone 3-hydroxylase, *VvF3h*, Vv_10003855; dihydroflavonol 4-reductase, *VvDFR*, Vv_10000485; leucoanthocyanidin dioxygenase, *VvLdox*, Vv_10000352) were significantly up-regulated in the infected samples compared with the healthy ones as shown by microarray and/or qRT-PCR analyses (Figures 1, 3B), [see Additional files 1, 3].

Among those genes related to the pathways which have been shown to possess crucial functions in mediating or orchestrating stress responses in plants, genes encoding salicylic acid carboxyl methyltransferase (VvSamt) that is responsible for the formation of methyl salicylate from salicylic acid and ethylene receptor (VvEtr) [21,22] were significantly up-regulated in the first examined growing seasons as demonstrated by qRT-PCR and microarrays (Figures 1, 3B), [see Additional file 1]. However, the expression of VvEtr was not significantly different from the phytoplasma-free samples in the second season. The transcript of the lipoxygenase gene (*VvLox*), which product may be involved in pest resistance or responses to wounding was significantly up-regulated in the first season, but only slightly induced in the second (Figures 1, 3B).

We found that the expression level of several genes related to heat stress and that of the gene encoding PR-10 were repressed in the samples of leaves with the phenotype 4 (Figure 3B), [see Additional file 3]. Four members of the WRKY family were significantly up-regulated in the infected leaves and additional five were significantly down-regulated (Figures 1, 3), [see Additional file 3]. Genes potentially involved in defense signal perception and MAPK-mediated signal transduction, transcriptional regulation, lignin biosynthesis, cell wall modification, and metabolism of reactive oxygen species were significantly differentially expressed in the samples with the phenotype 4 (Figure 3B). All these genes were expressed in a similar fashion as reported for grapevines infected with a fungus *Uncinula necator *[23], indicating a similar response of grapevine to biotic stress or a more general response of plants to pathogens.

Several MYB transcription factors were repressed upon 'BN' phytoplasma infection (Figure 3B). It has been suggested that the down-regulation of MYBs in cv. 'Chardonnay' is associated with a blockage of the lignin biosynthesis. As a consequence, an improper lignification of the young shoots as the most devastating symptom of the phytoplasma infection might follow [12].

### Changes in genes involved in hormone metabolism

Two genes involved in the cytokinin metabolism, specifically VvHP encoding histidine-containing phosphotransfer protein (*Vv_10001604*) involved in cytokinin signal transduction and VvCko encoding cytokinin oxidase (*Vv_10009579*), which plays a major role in the regulation of hormone levels in plants by irreversibly degrading cytokinins were differentially expressed in infected samples as shown by microarrays and the qRT-PCR assays (Figure 1) [see Additional file 3]. Similarly, several genes involved in synthesis, degradation and regulation of auxins and ethylene were significantly differentially expressed in healthy and infected samples [see Additional file 3]. Connection of these genes to phytoplasma infection has not been reported yet and will need further investigation.

## Discussion

Mechanisms of phytoplasma interaction with their hosts are largely unknown. Here we present a global insight into the physiological changes in the grapevine cv. 'Chardonnay' infected with 'BN' phytoplasma on the level of gene expression. To get a closer view of the processes which occur in nature, the analyses were performed in the field collected samples. Although similar studies are rare, they have already proven the importance of field experiments to evaluate complex correlations and to recognize inter-relationships among biotic and abiotic factors that structure ecosystems [11]. We investigated selected plants in two subsequent growing seasons. Unsurprisingly the phenotype expression was different in examined seasons, likely due to different concentration and distribution of 'BN' phytoplasmas in the host and/or climatic differences. Specifically, the growing season of 2004, when the strong leaf symptoms of the 'BN' phytoplasma infection developed, was colder and had more precipitation [see Additional file 4]. However, the overall effect of phytoplasma infection on the metabolic/signaling pathways remained similar in 2005 for the genes which showed the highest difference in expression. The only significant difference in the gene expression observed in the second growing season was a significant down-regulation of the gene encoding alcohol dehydrogenase 1, which was opposite as in the first growing season (Figure 1B). This might indicate its close association with symptom expression.

### Phytoplasma infection induces changes in carbohydrate metabolism

**Figure 4:**
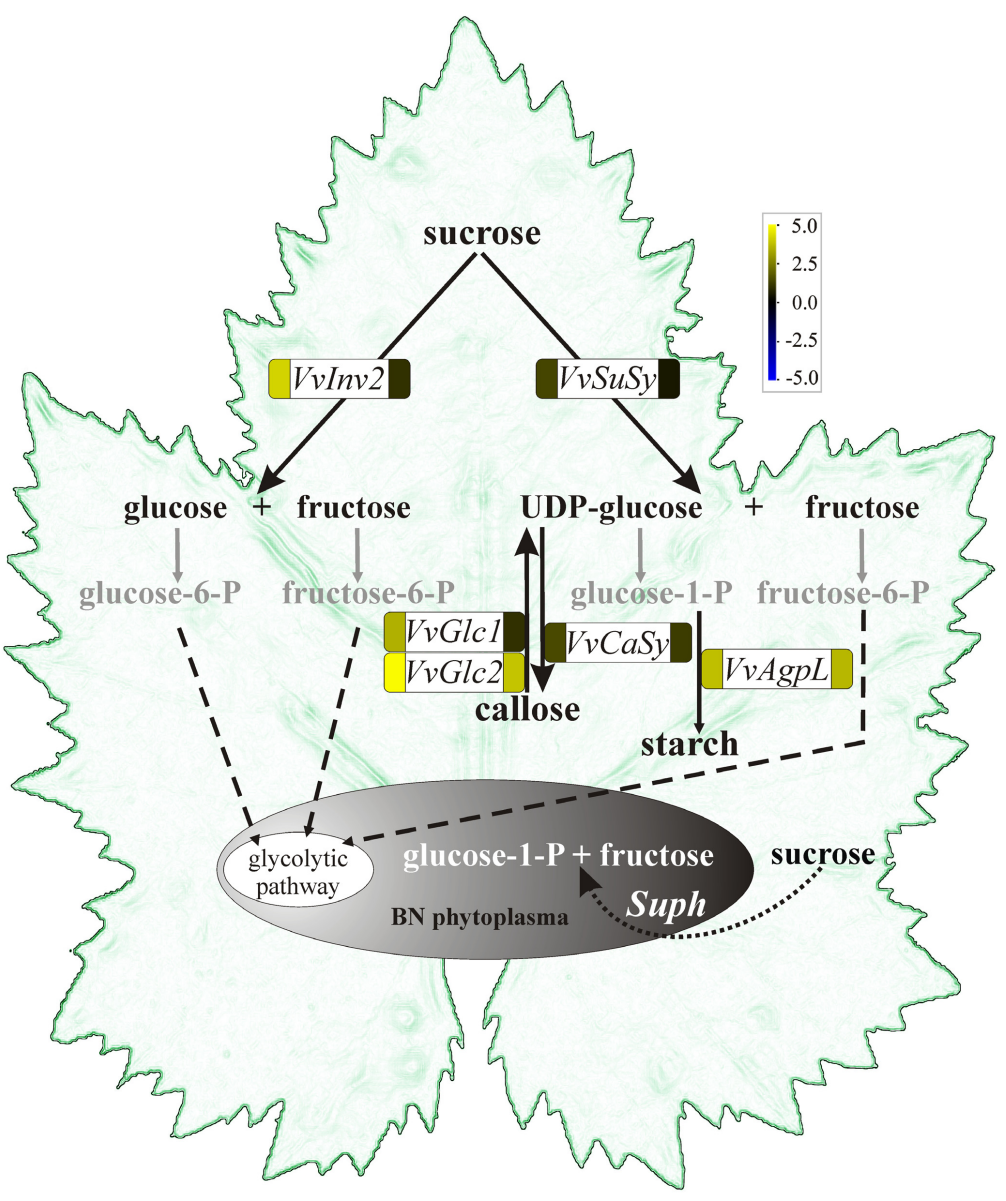
**A model of carbohydrate metabolism in phytoplasma infected grapevine tissues**. In addition, unlike the genomes of two strains of '*Ca*. P. asteris' [5,6] that of '*Ca*. P. australiense' has a complete gene for sucrose phosphorylase (*Suph*, [8]). The enzyme is important for sucrose cleavage into glucose-1-phosphate and fructose. It might be possible, therefore, that the 'BN' phytoplasma, which belongs to the same taxonomic stolbur group as '*Ca*. P. australiense', also possesses the gene for sucrose phosphorylase. How the enzyme degradation products might be further utilized by phytoplasmas is currently not known. Expression of genes that are represented in rounded rectangles was experimentally measured by qRT-PCR in both growing season (first growing season to the left and second growing season to the right of the gene symbol). Gene expression is color coded (yellow color represents up-regulation and blue color down-regulation in infected vs. healthy samples). *VvInv2 *encodes vacuolar acid invertase 2, *VvSuSy *encodes sucrose synthase, *VvGlc1 *and *VvGlc2 *encode two 1.3-β-glucanases, *VvCaSy *encodes callose synthase, *VvAgpL *encodes large subunit of ADP-glucose pyrophosphorylase and *Suph *encodes phytoplasmic sucrose phosphorylase.

A common effect of phytoplasma infection is accumulation of glucose, fructose, sucrose and starch in infected source leaves. They were considerably increased in leaves of *C. roseus *infected with several taxonomically unrelated phytoplasmas [14,17]. The starch and glucose content increased in the mature leaf material of papaya infected with '*Ca*. P. australiense' [15]. Similarly the soluble sugar and starch content increased in the leaves of coconut palm tree infected with coconut LY phytoplasma [16] and the amount of reducing sugars in maize leaves infected with maize bushy stunt phytoplasma [18]. Based on these observations, we herein explored at the gene expression level the assumption that phytoplasmas may alter the activity of plant enzymes involved in carbohydrate metabolism to meet their requirements for energy, growth and spread using the host plant's phloem system (Figure 4).

The major form of carbohydrate loaded into the phloem of photosynthetic source leaves is sucrose. Its up to 1 M concentration in the phloem sieve tubes is a potential food supply for phytoplasmas. A possible direct entrance of sucrose into the phytoplasma cell could be through ABC transporters used for importing sugars. The existence of genes encoding such transporters, including those for sucrose, has been revealed by comparison of known phytoplasma genomes [4,5,7]. It is worth noting that phytoplasmas lack any enzyme from the phosphotransferase system, which most bacteria use as an energy efficient way of simultaneously importing and phosphorylating sugars such as sucrose, glucose and fructose. In contrast, the genes needed to perform the glycolytic pathway are present [4,5,7]. Therefore, it appears that phytoplasmas depend on the uptake of phosporylated hexoses for their carbon source, which can then enter the phytoplasma glycolytic pathway [2]. The specific enzymes involved in the phosphorylation of adequate sugars in the grapevine leaves infected with 'BN' phytoplasma has not been evaluated yet. However, genes for several enzymes involved in the interconversions between sugar phosphorylated forms, specifically phosphoglucomutase, phosphoglucose isomerase, pyrophosphate-dependent phosphofructokinase, fructose 1.6-bisphosphate aldolase, glyceraldehyde-3-phosphate dehydrogenase and phosphoglycerate kinase, were identified as up-regulated in the infected samples [see Additional file 3]. On the contrary, genes encoding fructose 1.6-bisphosphate aldolase and glyceraldehyde-3-phosphate dehydrogenase were found as repressed in the 'Chardonnay' plants infected with 'BN' phytoplasma [12]. Additional research is required to explore if the products of the corresponding enzyme reactions might enter and be utilized upstream of the phytoplasma glycolytic pathway.

The only known enzymes of sucrose cleavage in plants are invertases, which produce glucose and fructose; and sucrose synthase, which produces UDP-glucose and fructose [24]. Two vacuolar acid invertase genes (i.e. *VvInv1 *and *VvInv2*) in grapevine encode translation products that are 62% identical to each other but have different expression patterns [25]. *VvInv1 *is expressed predominately in berry skin and flesh. In addition to its expression levels in young leaves, the *VvInv2 *expression is the highest in young flowers, with lower expression level in berries and no expression detected with Northern blot analysis of its mRNA in fully expanded leaves [25]. Accordingly, in the midrib of phytoplasma-free expanded leaves we did not detected any transcripts of *VvInv1 *or *VvInv2 *with microarrays in the first growing season. Moreover, the level of transcript of *VvInv2 *estimated with qRT-PCR was also extremely low (Figure 1A). On the other hand, the transcript corresponding to *VvInv2 *was significantly increased in the midribs of the infected leaves in the first growing season and was up-regulated in the second one (Figure 1), [see Additional file 3]. Interestingly, in *C. roseus *and tomato infected with stolbur phytoplasmas, no differential expression of the vacuolar invertase gene was observed in leaf tissue, but its enzymatic activity was increased [26].

### Callose deposition in grapevine infected with 'BN' phytoplasma

In the leaf midrib of the infected samples a transcript of the sucrose synthase gene *VvSuSy *was significantly up-regulated in the first season and slightly induced in the second one (Figure 1). It has been recently demonstrated that in phloem, sucrose synthase is localized in both companion cells and sieve elements [24]. The sieve tube localization could facilitate sucrose synthase's role in directly supplying UDP-glucose for the rapid biosynthesis of callose plugs in the sieve pores. To support this hypothesis, a significant induction of callose synthase transcript was observed in symptomatic samples. A high UDP-glucose concentration has been reported in phloem exudates from an apple tree infected with phytoplasma-like organism [27]. In addition, a physical obstruction of the phloem sieve tubes by callose deposition has been demonstrated in the sieve elements of *C. roseus *and *Euphorbia pulcherrina *infected with phytoplasmas [28]. Necrosis and the collapse of sieve elements that follow [2] might be associated with reduced carbohydrate translocation, their accumulation in mature leaves and decreased starch content in sink tissues of phytoplasma infected plants [14-18]. Callose deposition is a dynamic process coordinated through the activities of callose synthase and the callose hydrolyzing enzyme 1,3-β-glucanase. Based on observations that glucanase activities are enhanced under conditions promoting callose accumulation, a short life span of callose molecules, shifted towards catabolism, has been suggested [29]. It is noteworthy in this regard that the transcriptional analyses showed a strong increase in transcription of 1,3-β-glucanase genes (*VvGlc*) also in grapevine (Figure 1), [see Additional file 3]. Besides catalyzing the hydrolysis of 1,3-β-linkages of cell-wall polymers found in plants, certain 1,3-β-glucanases also hydrolyze fungal cell-wall 1,3/1,6-β-glucans and are induced when plants are infected by microbial pathogens and/or herbivores [30-32]. Therefore, they are referred to as pathogenesis-related protein from class 2 (PR-2). At the moment, we could not rule out the possibility that the increased gene transcription of β-1,3-glucanase in grapevine is due to the phytoplasma infection, in order to make callose degrading products utilizable for phytoplasmas and/or to facilitate their spread through the plant, as it has been suggested for the planthopper attack [32] or spread of plant viruses [30]. The absence of typical phytoplasma leaf symptoms in the second growing season was correlated with an insignificant differential expression of genes encoding sucrose synthase, callose synthase and 1,3-β-glucanse gene *VvGlc3*.

High accumulation of the ADP-glucose pyrophosphorylase gene transcript (*VvAgpL*) (Figure 1) correlates well with biochemical measurements of the increased starch level in leaves of different host plants infected with different phytoplasma species [14-18].

### Grapevine photosynthesis genes responding to 'BN' phytoplasma

There is growing evidence that several steps in photosynthesis are repressed during phytoplasma infection and it is generally believed that a feedback inhibition of photosynthesis causing chlorosis is a result of accumulating carbohydrates in source leaves [2]. However, it is not clear if the observed alterations are directly linked to phytoplasmas presence or, as proposed by Bertamini et al. [33,34], are non-specific, general responses to the infection. Nevertheless, our results here provide new insights on the photosynthesis associated proteins in phytoplasma infected plants at the transcriptome level, and thus complement already obtained biochemical data from studies on *C. roseus *infected with ash yellows phytoplasmas, on apple trees infected with phytoplasmas causing apple proliferation and grapevine infected with 'BN' phytoplasma [33,34].

Based on the indirect measurement of the variable fluorescence yield together with the loss of several thylakoid polypeptides, it has been proposed that in phytoplasma infected leaves, it is mainly photosystem II (PSII) which is impaired, particularly at the donor site [33,34]. Our results support this hypothesis. Eleven genes encoding chlorophyll a/b-binding proteins in the PSII and three in PSI were significantly down-regulated (Figure 3A), [see Additional file 3]. It has been shown that their synthesis is specifically repressed by free hexoses [35], whose accumulation in leaves is generally associated with phytoplasma infections [14-18]. Significant repression of the *VvRbcL *gene encoding Rubisco large subunit (Figure 3A) is consistent with the protein loss reported by [33,34] in infected grapevine and apple trees. It is not evident from the biochemical studies of net photosynthesis rate in phytoplasma infected *C. roseus *[36], if its reduction was due to the lower activation state of Rubisco or lower Rubisco content. However, the grapevine transcriptome results suggest that both factors contribute to the reduced photosynthesis, since genes for Rubisco large subunit (*VvRbcL*) and Rubisco activase (*VvRca*) were significantly down-regulated in infected plants (Figure 3A). Similar suppression of those transcripts were also observed after the flea beetle attack of *Solanum nigrum *[11] and after PVY infection of *Solanum tuberosum *[37], suggesting a more general effect of the biotic stress. Comparison of asymptomatic and symptomatic samples showed that in the electron transport pathway the expression of genes involved in cytochrome *b6f *complex decreased in the infected leaves (Figure 3A), [see Additional file 3]. This finding is directly correlated with the observed down-regulation of genes encoding ATP synthase (Figure 3A), [see Additional file 3] and in accordance with the biochemical model of photosynthesis in which the regeneration of ribulose bisphosphate is dependent on the rate of electron transport required for the generation of energy and reducing equivalents ATP and NADPH. Recently published results on differentially expressed genes involved in photosynthesis of cv. 'Chardonnay' infected with 'BN' phytoplasma support our data [12].

### Differentially expressed genes involved in secondary metabolism upon 'BN' phytoplasma infection

Genes for the key enzymes in a flavonoid biosynthetic pathway, which leads to the synthesis of flavonols, anthocyanins and condensed tannins [38] were mostly up-regulated in the symptomatic samples in comparison with the asymptomatic ones (Figure 3B), [see Additional file 3]. The increase in transcripts of dihydroflavonol 4-reductase (*VvDfr*) and leucoanthocyanidin dioxygenase *VvLdox *[see Additional file 3] has also been shown in *Populus tremuloides *after wounding or herbivore attack [39], and in grapevine leaves and hybrid poplar after fungal infection [40,41]. To the best of our knowledge the results presented here are the first report of the up-regulation of *Ldox *in bacterial infected plants. Supporting data for the assumption of correlation between the phytoplasma infection and an up-regulation of a *VvLdox *gene include the *VvLdox *transcript increase in developing grape berries and young grapevine leaves, but its substantial drop in the mature leaves [38].

### Induction of osmotin and thaumatin- like proteins upon 'BN' phytoplasma infection

Among several other PR protein classes, the significant up-regulation of genes encoding thaumatin-like and osmotin pathogenesis-related proteins from group 5 (PR-5) was shown in the infected leaves (Figures 1, 3B), [see Additional file 3]. Their presence seems ubiquitous during the development of grapevine berries [42-44]. In addition, both osmotin and thaumatin-like proteins or the transcripts of their genes were found in grapevine leaves and berries infected with fungi *Uncinula necator*, *Phomopsis viticola *and *Botrytis cinerrea *[23,43]. Accumulation of PR-5-like proteins was also reported in 'Chardonnay' grapevine infected with 'BN' phytoplasma [12] and *Chrysanthemum coronarium *infected with onion yellows phytoplasma [45]. It has been suggested that these proteins play an important role in protection against fungal pathogens and possibly other stresses [44].

## Conclusion

Our field conducted study revealed complex interactions among grapevine cv. 'Chardonnay' and 'BN' phytoplasma and thus substantially contribute to the understanding of still largely unknown mechanisms of phytoplasma patogenicity. The obtained results indicate that the phytoplasma infection induced both, the reprogramming of the primary metabolic pathways, as well as the activation of the secondary ones, possibly related to the defense mechanisms, and thus support the hypothesis that metabolic reprogramming is one of the plants' defense strategies against their pathogens [46]. The gene expression changes in response to infection by phytoplasmas may support phytoplasma nutrition by promoting alterations in host's sugar metabolism. In addition, the induction of defense related pathways is in line with the hypothesis that defense compounds are induced in resistant as well as in susceptible interactions, with the main difference being in the speed and intensity of the response [23]. The outcome of this study will likely impact not only the fundamental aspects of grapevine interactions with its pathogens, but also grape improvement, for example by yielding marker genes that can be used in new diagnostic assays for phytoplasmas or by identifying candidate genes that contribute to the improved properties of grape (e. g. disease resistance).

## Methods

### Plant material, ELISA tests for viruses and RNA extraction

The study was carried out in a production vineyard of grapevine (*Vitis vinifera *L.) cv. 'Chardonnay' in the south-western part of Slovenia in the Brda region (45°58' N, 13°32' E). The vineyard was regularly treated with fugicides against *Plasmopara viticola*, *Erysiphe necator *and *Phomopsis viticola*. The leaf samples of 26 field grown grapevine plants were collected at the véraison stage of grape berry development on August 5^th ^2004 when pronounced symptoms of grapevine yellows (GY) were observed on the plants. The same plants were additionally sampled on August 9^th ^2005. The weather conditions in both growing seasons are shown in [Additional file 4].

All sampled plants were monitored for infection with other pathogens. They were tested for virus presence using DAS-ELISA for GFLV, GVA, GLRaV-1, GLRaV-2, GLRaV-6 and GFkV antibodies (Bioreba) and using PTA-ELISA for GVB (Agritest) according to the producer instructions. Optical density of all samples was measured at 405 nm. ELISA reads were considered positive, when they reached values higher than 2-fold of the value of the negative controls [see Additional file 1].

All samples represented midribs of leaves with central veins, where phytoplasmas are presumably the most abundant. Leaf tissue pieces with 1-2 mm of lamina on each side of the midrib were cut in the field and immediately stored in liquid nitrogen. Each sample comprised central midribs of three youngest fully developed leaves from the same shoot. All samples were collected from 1-2 m above ground between 10 and noon on the same day. See [Additional file 5] for a schematic representation of the vineyard. In the first season 25 samples from shoots exhibiting typical phytoplasma-related symptoms and 27 samples from asymptomatic shoots were collected. Leaf samples were sorted according to their expressed phenotype scaled from zero to four. Zero stage phenotype showed no changes in phenotype; stage one showed extremely slight yellowing; stage two slight yellowing; stage 3 prominent yellowing; and samples with a phenotype four had typical phytoplasma related symptoms: yellowing, downward curling leaf laminas, bristle leaf laminas. In the next season the same plants were sampled.

Total RNA was extracted using RNeasy Plant Mini Kit (Qiagen), treated with DNaseI (Invitrogen), quantified spectrophotometrically (Nanodrop, Nanodrop Technologies) and quality checked using Bioanalyser (Agilent). Presence of 'BN' phytoplasma was checked and quantified using qRT-PCR on cDNA (as described in [13,47]) in each sample.

### Microarray hybridization, data analysis and visualization

RNA isolated from samples collected only in the first season was pooled to create 4 infected and 4 healthy groups according to the similarity of expression of genes encoding sucrose synthase, alcohol dehydrogenase 1 and Hsp70 determined by qRT-PCR in each sample [see Additional file 6] for a general experimental design. Those genes have been determined in our previous study as significantly differentially expressed in healthy or infected samples [13]. Common reference design was selected for microarray hybridizations. A fraction of total RNA of each sample was pooled to create a common reference pool which was further processed in the same way as the rest of the RNA samples.

For microarray hybridizations 70 mer oligonucleotide microarrays were used containing all 14562 oligonucleotides from the AROS V1.0 OligoSet (Operon), printed in one replica onto silver coated slides (Amersham). Total RNA was cleaned up using High Pure RNA Tissue Kit (Roche) according to the manufactures' instructions and spectrophotometrically quantified. Antisense RNA (cRNA) amplification and simultaneous Cy3- and Cy5-labelling were performed as described in [48] with the following modification: hybridization mixtures were prepared by combining 10 μg of Cy-5 labeled sample cRNA and 10 μg of Cy-3 labeled common reference cRNA. Immediately prior to hybridization, 60 μl of cRNA mixture, 60 μl of 4× hybridization buffer (Amersham) and 120 μl of deionized formamide were combined, vortexed, denatured at 95°C for 5 min and cooled 3 min on ice. Microarrays were placed into the Amersham Lucidea hybridization station (Amersham) and hybridized with hybridization mixtures containing 20 μg of labeled cRNA at 37°C for 16 hours. Microarray slides were washed in three successive steps: i) 20 min with 1× SSC and 0.2% SDS, ii) 10 min with 0.1× SSC, 0.2% SDS (twice), iii) 10 min with 0.1× SSC. All washing solutions were filtered through 0.2 μm filters and heated to 37°C. Microarray slides were finally washed with isopropanol, air-dryed and scanned with Axon Genepix 4000B (Axon Instruments) on the same day, as described in [48]. All microarrays were visually inspected and all spots with poor morphology were flagged to be left out from further analysis. Microarray experimental design details are presented in [Additional file 7]. Microarray platform was submitted to Gene Expression Omnibus (GEO, , Platform GPL6637).

MA plots (M representing the intensity ratio of both Cy-channels (y-axis) and A the average intensity of both Cy-channels (x axis) for every probe on the microarray), boxplots and surface plots of microarray data showed that the quality of the data was consistent across all microarrays and could therefore be used in further data analysis. All microarray data was deposited to the NCBI's Gene Expression Omnibus, a public gene expression/molecular abundance repository (DataSet accession number: GSE10906).

Data analysis procedures were performed in R environment for statistical computing [49]. Package *limma *[50] was used for importing microarray data from GenePix files, for data pre-processing (background correction and normalization) and to search for differentially expressed genes between infected and healthy grapevine samples using linear models (common reference design). An approach combining two normalization methods (*loess *and *vsn*) and four types background subtraction (*none*, *subtract*, *half *and *normexp*), thus yielding eight pre-processing methods, each leading to its set of differentially expressed genes, was used (described in [51]). The final list of differentially expressed genes was obtained as an intersection of lists of top differentially expressed genes (P-values < 0.05) resulting from six pre-processing combinations (the two most divergent were omitted as unreliable for analysis of this dataset). MA plots, boxplots and surface plots for raw and normalized data for each microarray were prepared to check the quality of raw data and the effect of normalization.

The final list of differentially expressed genes was imported into the MapMan visualization tool [20,52] where genes are organized in graphically represented metabolic pathways and the corresponding log2-fold change for each gene is color coded. Log2-fold change represents the difference in level of gene expression between infected and healthy samples.

### Real-time PCR

Publicly available sequences of transcripts from GenBank^® ^and DFCI Grape Gene Index databases were analyzed in the set-up of quantitative real-time PCR reactions (qRT-PCR), with SYBRGreen I and TaqMan^® ^chemistry for 15 target genes, selected from the list of differentially expressed genes obtained from the microarray data analysis: vacuolar acid invertase 2 (*VvInv2*), ADP-glucose pyrophosphorylase (*VvAgpL*), apocytochrome f precursor (*VvAcyt*), ethylene receptor (*VvEtr*), flavanone 3-hydroxylase (*VvF3h*), histidine-containing phosphotransfer protein involved in cytokinine signal transduction (*VvHP*), lipoxygenase (*VvLox*), osmotin-like protein (*VvOlp*), S-adenosyl-L-methionine:salicylic acid carboxyl methyltransferase (*VvSamt*), WRKY54 (*VvWrky*), callose synthase (*VvCaSy*), cytokinin oxidase (*VvCko*), β-1,3-glucanase I (*VvGlc1*), β-1,3-glucanase II (*VvGlc2*) and β-1,3-glucanase III (*VvGlc3*). Primer Express^© ^software (Applied Biosystems) was used for the design of primer pairs. Specificities of the designed amplicons were tested *in silico *using BLASTn search of public databases [53]. Primer pairs were designed in the region of microarray oligo design to ensure the compatibility of results between both platforms. Details of the primer design are described in [Additional file 8].

Two amplicons published by [15], i.e. sucrose synthase (*VvSuSy*) and alcohol dehydrogenase I (*VvAdh1*)) were added to the set of 15 newly designed amplicons for sample screening. All qRT-PCR reactions were performed on an ABI PRISM^® ^7900 HT Sequence Detection System (Applied Biosystems) as described in [13].

The relative quantification approach was used essentially as described in [54] and [13] with cytochrome oxidase (Cox; [55]) and 18S (Eukaryotic 18S rRNA TaqMan endogenous control, Applied Biosystems) as endogenous controls required for the normalization process. Due to low expression values of genes *VvSamt *and *Vvinv2 *in samples from healthy leaves (Ct values were above 34 or even undetermined in the higher of the two dilutions used in qRT-PCR reactions). Therefore we replaced the Ct values in all healthy samples that had Ct values above 34.0 with values of 34.0 for the higher and 30.7 for the lower dilution in order for the samples to pass the quality control and to be included into the t-statistics. This procedure is common in the pre-processing of microarray data where negative signal values after background correction (low signal intensity spots) are corrected with an arbitrary offset [56]. In this way, genes with low expression values in a certain state (in our example in healthy samples) are not excluded from analysis since they may be highly expressed in another state and may be therefore differentially expressed.

The Welch two sample t-test was used to determine statistically significant differences between relative expression ratios of infected and healthy samples with a P = 0.05 as the limit for statistical significance.

### Sample classification using gene expression data

Samples collected in the field in two subsequent growing seasons were classified into two groups based on the already analyzed qRT-PCR gene expression data of 17 genes with support vector machines algorithm (SVM, [57]). Gene expression data were first scaled, separately for each gene, by the subtraction of median gene expression of healthy samples and variance stabilized by the division with variance of gene expression of healthy samples. Prior to classification, an algorithm was trained on the data where healthy samples were identified. Classification produced two groups of samples, which were visualized as heat maps. SVM algorithm was performed in MultiExperiment Viewer v4.2 software (, [58]).

## Authors' contributions

MH participated in the design of the study, collected the samples, carried out the microarray and real-time PCR studies and helped to draft the manuscript. PN participated in the real-time PCR studies; AR and AB participated in the statistical analysis; NT participated in the microarray studies; MR, KG and MD elaborate the study, participated in its design and completion; KG helped to draft the manuscript. MD coordinated the study and drafted the manuscript. All authors read and approved the final manuscript.

## Supplementary Material

Additional file 1 **Plant disease status and differential expression of selected genes (*VvvInv2*, *VvAcyt*, *VvAgpL*, *VvHP*, *VvEtr*, *VvF3h*, *VvLox*, *VvOlp*, *VvSam*t, *VvWrky*, *VvAdh1*, *VvSuSy*, *VvCaSy*, *VvCko*, *VvGlc1*, *VvGlc2 *and *VvGlc3*) in the first and second growing season determined by qRT-PCR**. The data provided represent all tested samples with their phenotypes, a presence of phytopasma and viruses and a differential expression of 17 selected genes. Click here for file

Additional file 2 **Typical symptoms of grapevine Yellows 'Bois noir' on a 'Chardonnay' grapevine plant in a production vineyard**. This panel shows leaf curling and discoloration of leaf veins and laminas and interveinal yellowing with berry withering. Click here for file

Additional file 3 **List of differentially expressed genes in the first growing season determined by microarray analysis**. In a spread sheet A is a list of all differentially expressed genes, and in a spread sheet B is a list of genes that are discussed in the paper. Click here for file

Additional file 4 **The average temperature and precipitation in the area of the examined vineyard during growing seasons of 2004 and 2005**. The data provided show weather data obtained from a meteorological station positioned around 10 km SE from the vineyard. Click here for file

Additional file 5 **A schematic view of the vineyard in which the samples were collected**. The picture shows the position of all sampled plants and their disease status in the scheme of tested plot. Click here for file

Additional file 6 **Experimental design of the study**. The picture shows the complete workflow including qRT-PCR, microarrays and data analysis. Click here for file

Additional file 7 **Microarray experimental design**. Summary of microarray experimental design. Click here for file

Additional file 8 **Real-time PCR design**. The protocols of amplicon design for use with SYBRGreen I chemistry are shown for 17 representative genes. Click here for file
