# Supplementary material for: 'Bois noir' phytoplasma induces significant reprogramming of the leaf transcriptome in the field grown grapevine
Source: BMC Genomics. 2009 Oct 2;10:460. doi: 10.1186/1471-2164-10-460 (PMC2761425; doi:10.1186/1471-2164-10-460)
Supplement: Additional file 2 — Typical symptoms of grapevine Yellows 'Bois noir' on a 'Chardonnay' grapevine plant in a production vineyard. This panel shows leaf curling and discoloration of leaf veins and laminas and interveinal yellowing with berry withering. [file 1471-2164-10-460-S2.DOC]

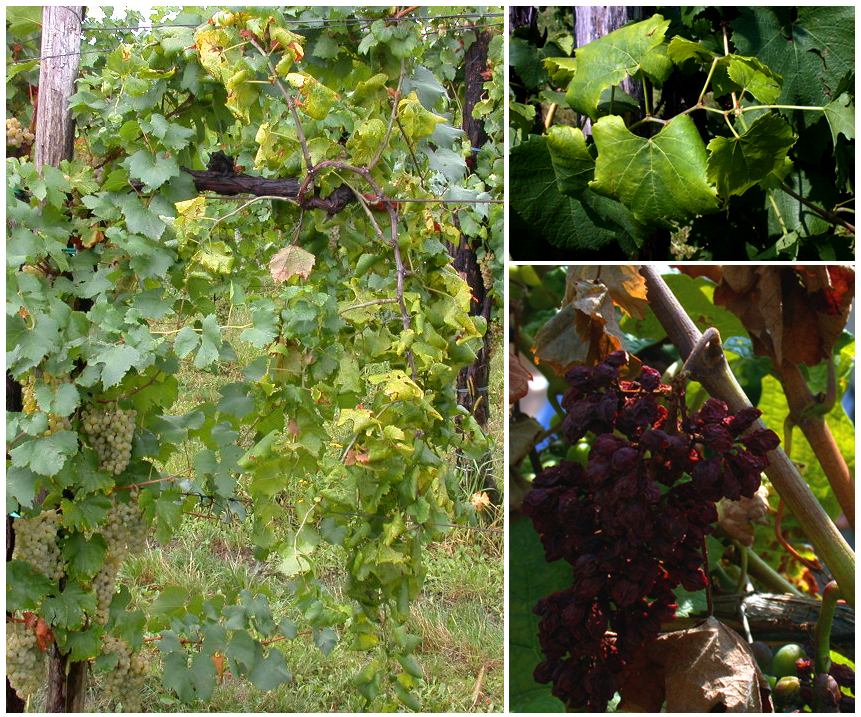


Additional file 2: **Typical symptoms of Grapevine Yellows ‘Bois noir’ on a ‘Chardonnay’ grapevine plant in a production vineyard.**

Symptoms include leaf curling and discoloration of leaf veins and laminas and interveinal yellowing. Leaves have a hard, brittle texture. Berry withering can also occur. Top right image represents phytoplasma-infected sample ID 34 in August 2004. See Additional file 5 for location of the plant.
