# Supplementary material for: 'Bois noir' phytoplasma induces significant reprogramming of the leaf transcriptome in the field grown grapevine
Source: BMC Genomics. 2009 Oct 2;10:460. doi: 10.1186/1471-2164-10-460 (PMC2761425; doi:10.1186/1471-2164-10-460)
Supplement: Additional file 4 — The average temperature and precipitation in the area of the examined vineyard during growing seasons of 2004 and 2005. The data provided show weather data obtained from a meteorological station positioned around 10 km SE from the vineyard. [file 1471-2164-10-460-S4.DOC]

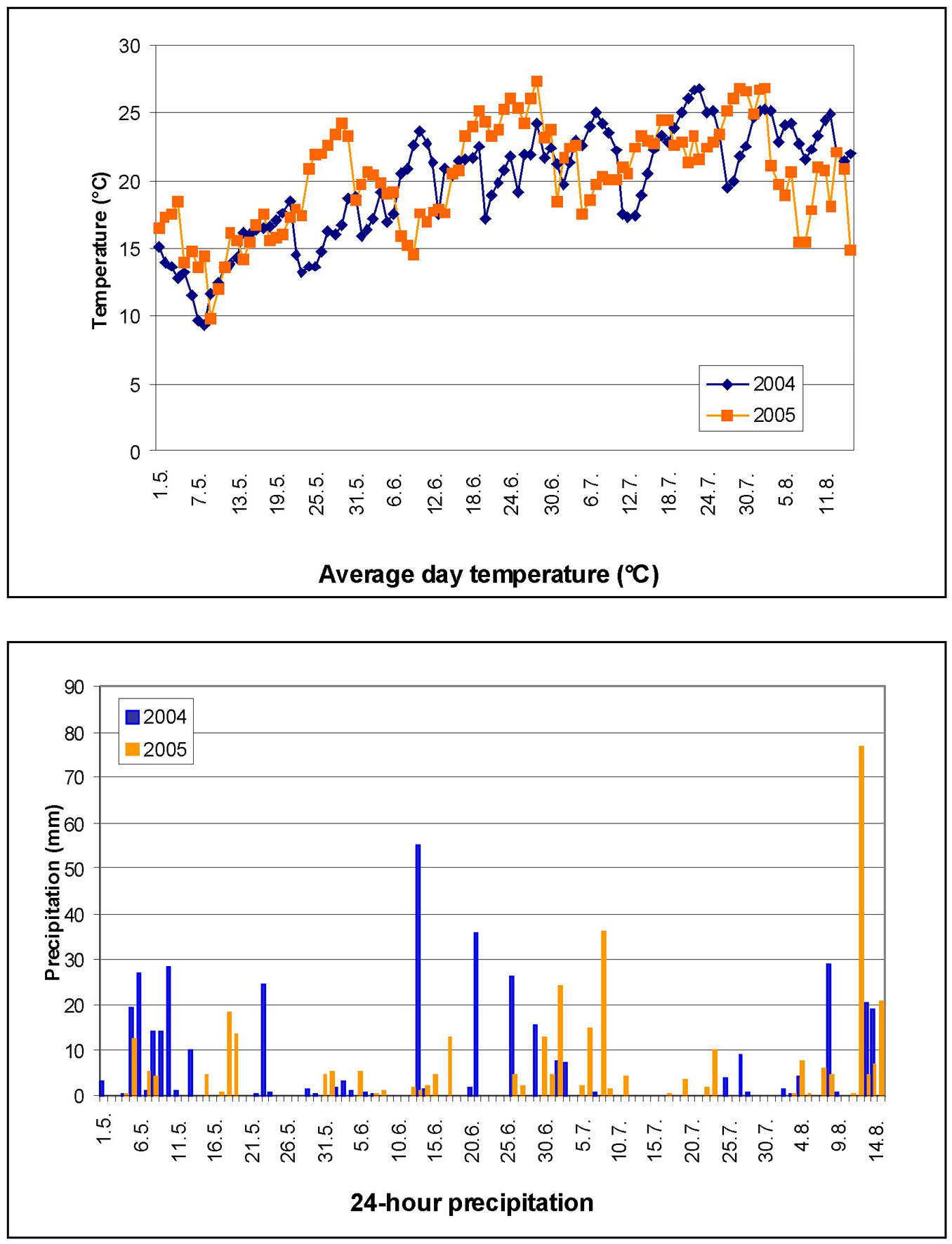


Additional file 4: **The average temperature and precipitation in the area of the examined vineyard during growing seasons of 2004 and 2005.**

Weather conditions are obtained from a meteorological station positioned around 10 km SE from the vineyard.
