# Supplementary material for: 'Bois noir' phytoplasma induces significant reprogramming of the leaf transcriptome in the field grown grapevine
Source: BMC Genomics. 2009 Oct 2;10:460. doi: 10.1186/1471-2164-10-460 (PMC2761425; doi:10.1186/1471-2164-10-460)
Supplement: Additional file 5 — A schematic view of the vineyard in which the samples were collected. The picture shows the position of all sampled plants and their disease status in the scheme of tested plot. [file 1471-2164-10-460-S5.DOC]

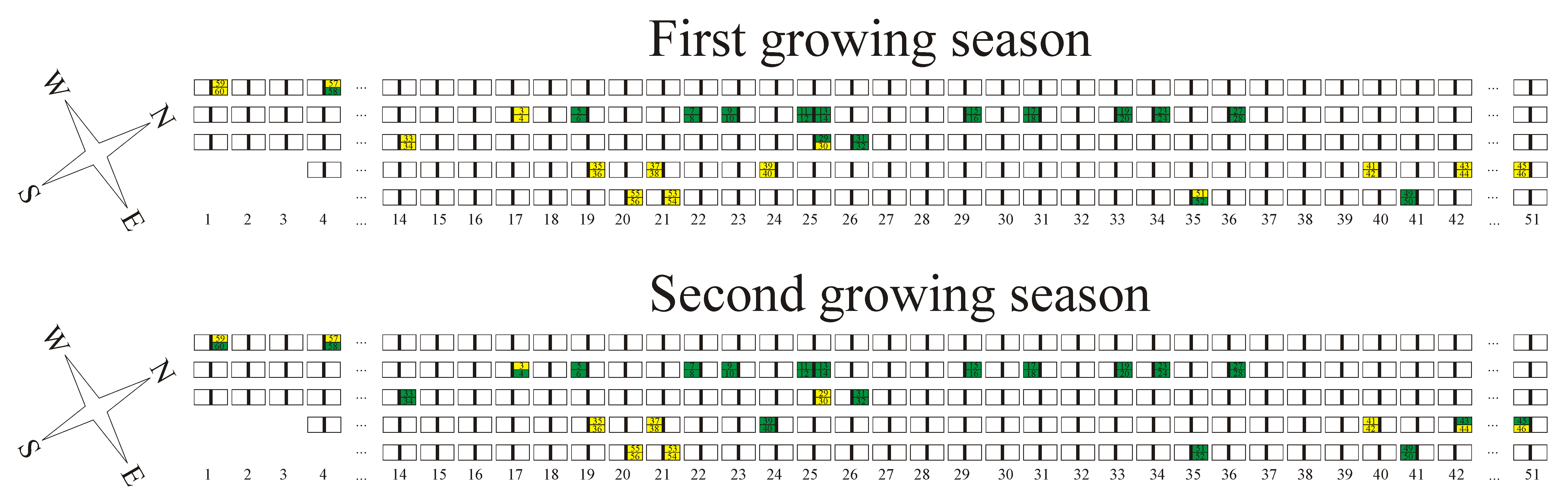


Additional file 5: **A schematic view of the vineyard in which the samples were collected.**

Each subunit of the image (four squares and a thick black line in the middle) represents two plants, one growing on the left side of the pole and the second on the right side of the pole. Since two shoots were sampled per plant one plant is represented with two rectangles on top of each other. Therefore each small rectangle corresponds to a single sample, which is depicted with a sample ID. Disease status of individual sample’s shoot is color coded (yellow – infected, green – healthy sample). The two collection seasons are presented in separate schemes.
