# Supplementary material for: 'Bois noir' phytoplasma induces significant reprogramming of the leaf transcriptome in the field grown grapevine
Source: BMC Genomics. 2009 Oct 2;10:460. doi: 10.1186/1471-2164-10-460 (PMC2761425; doi:10.1186/1471-2164-10-460)
Supplement: Additional file 6 — Experimental design of the study. The picture shows the complete workflow including qRT-PCR, microarrays and data analysis. [file 1471-2164-10-460-S6.DOC]

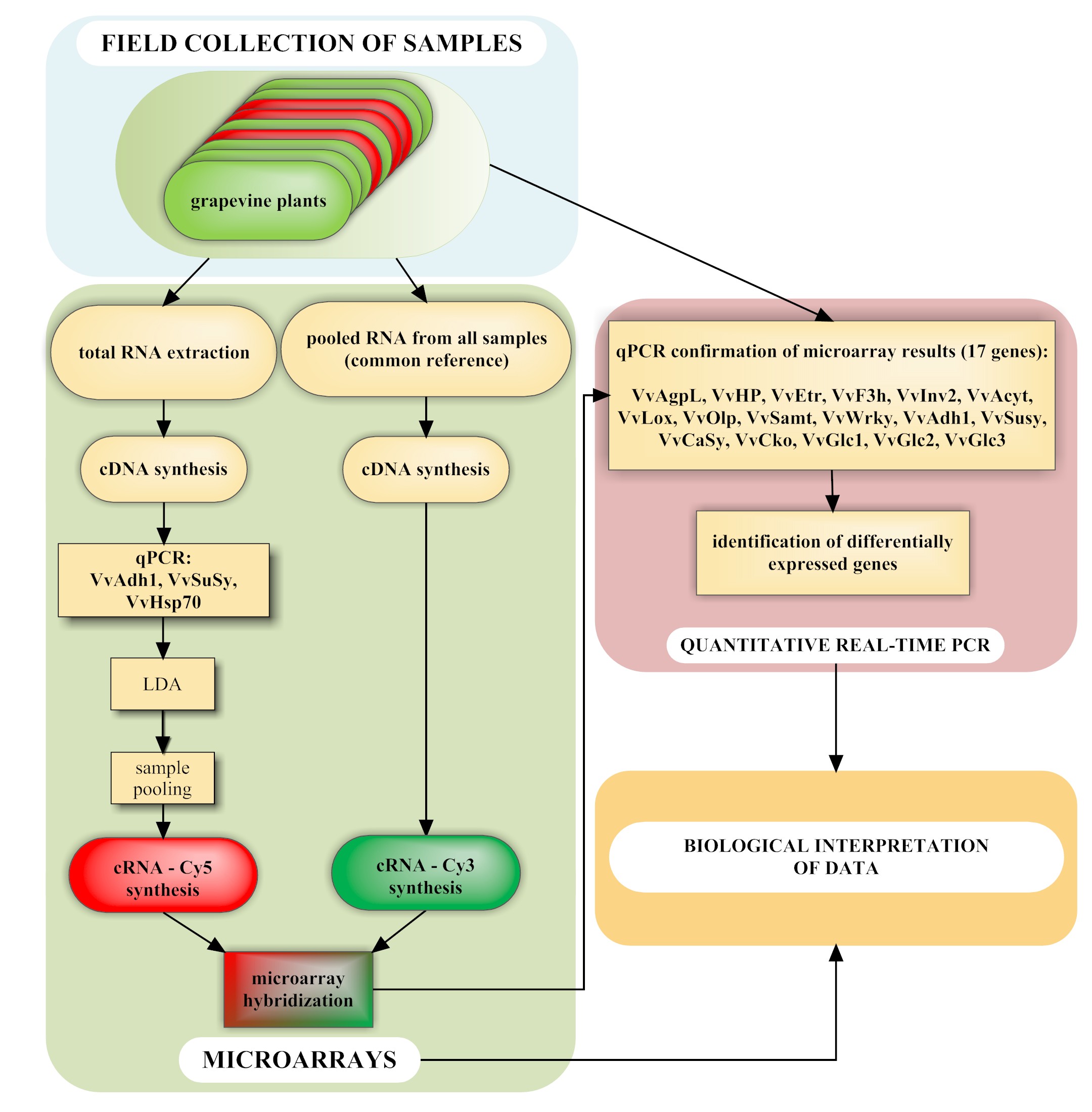


Additional file 6: **Experimental design of the study**

A complete workflow including qRT-PCR, microarrays and data analysis is shown. See Additional file 7 for details on the microarray experimental design.
