# Supplementary material for: 'Bois noir' phytoplasma induces significant reprogramming of the leaf transcriptome in the field grown grapevine
Source: BMC Genomics. 2009 Oct 2;10:460. doi: 10.1186/1471-2164-10-460 (PMC2761425; doi:10.1186/1471-2164-10-460)

Additional file 7: **Microarray experimental design**

1. Leaf tissue pieces with 1-2 mm of lamina on each side of the midrib were cut in the field and immediately stored in liquid nitrogen. Each sample was comprised from central midribs of the youngest three leaves with complete morphology on the same shoot.
2. Samples were collected from symptomatic plants and non-symptomatic plants.
3. Total RNA was extracted and reverse transcribed.
4. All samples were tested with qRT-PCR for the presence of bois noir phytoplasma. Symptomatic samples that contained phytoplasma locally were considered to be infected. Non-symptomatic samples that did not contain phytoplasma locally were considered to be healthy.
5. qRT-PCR screening for gene expression of sucrose synthase (SuSy), alcohol dehydogenase 1 (Adh1) and Hsp70 was performed on all samples followed by linear discriminant analysis (LDA) [19].
6. Four samples of healthy and four samples of infected samples were created for microarray hybridizations. In case of healthy samples each was created by pooling equal amounts of total RNA of three to four healthy samples that were grouped closely according to the LDA analysis of gene expression profile of SuSy, Adh1 and Hsp70. The same procedure was applied for infected samples. Individual samples used for pooling are presented in the table below.

| **Microarray pool** | **Samples used for pooling** |
| --- | --- |
| Healthy pool 1 | 1, 17, 28 |
| Healthy pool 2 | 16, 58 |
| Healthy pool 3 | 14, 23, 26, 27 |
| Healthy pool 4 | 7, 19, 29 |
| Infected pool 1 | 39, 43, 45 |
| Infected pool 2 | 41, 56, 59 |
| Infected pool 3 | 46, 53, 54 |
| Infected pool 4 | 3, 4, 30 |

1. Common reference RNA was created by pooling a fraction of total RNA all samples.
2. Total RNA was purified and subjected to a process of amplification and simultaneous labeling with Cy-dyes [56]. RNA from samples was labeled with Cy-5 and reference RNA with Cy-3.
3. Each microarray hybridization was performed by combining 10 μg of Cy-5 labeled sample RNA and 10 μg of Cy-3 labeled reference RNA onto a 70 mer oligonucleotide microarray resulting in a common reference design. Four such hybridizations were prepared for healthy samples and four for infected samples representing four biological replicates for each disease state.

Schematic representation of microarray experimental design.


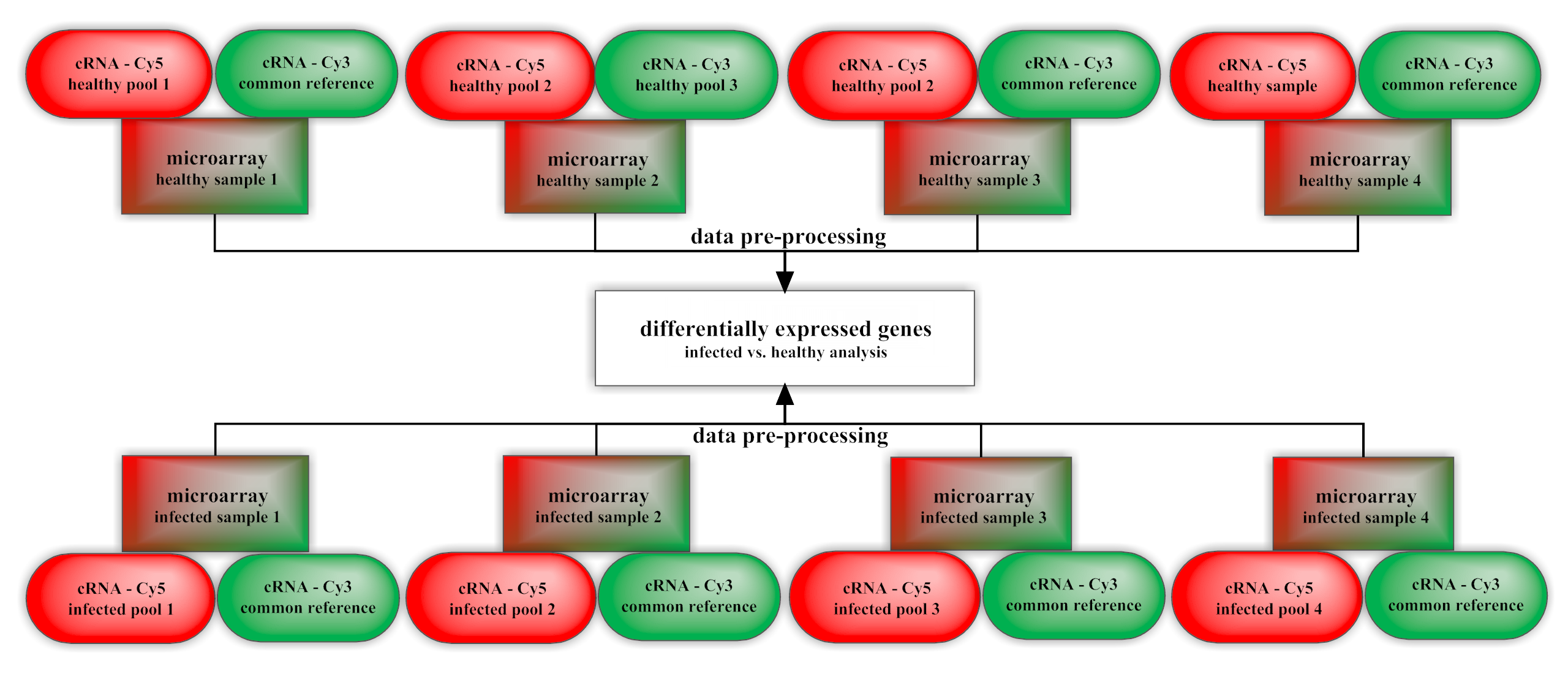

Supplement: Additional file 7 — Microarray experimental design. Summary of microarray experimental design. [file 1471-2164-10-460-S7.DOC]
