# Supplementary material for: 'Bois noir' phytoplasma induces significant reprogramming of the leaf transcriptome in the field grown grapevine
Source: BMC Genomics. 2009 Oct 2;10:460. doi: 10.1186/1471-2164-10-460 (PMC2761425; doi:10.1186/1471-2164-10-460)
Supplement: Additional file 8 — Real-time PCR design. The protocols of amplicon design for use with SYBRGreen I chemistry are shown for 17 representative genes. [file 1471-2164-10-460-S8.DOC]

Additional file 8: **Real-time PCR design**

Amplicons for 17 representative genes have been designed for use with SYBRGreen I chemistry (Table 1). BLAST search of public databases showed no unspecific hits with high similarity to the amplicon sequences (E < 0·04). Melting curve analysis for all amplicons detected no unspecific products or primer-dimer formation during amplification except in the case of *VvInv2* and *VvSamt* (results not shown); therefore TaqMan® probes were additionally designed for these two amplicons in order to ensure the specificity of target amplification. TaqMan® probes were also used in the case of *VvCaSy*, *VvCko*, *VvGlc1*, *VvGlc2* and *VvGlc3*. All amplicons were validated on serial dilutions of seven samples (in duplicate wells, dilutions ranging from 10-fold to 10^5^-fold). Performance characteristics (slope, efficiency and correlation coefficient between duplicate samples) were appropriate for reliable quantitative analysis (Table 2). Validation data was used to select the most appropriate dilutions of cDNA samples for each gene in order to obtain Ct values ranging from 22 to 34.

**Table 1**. Primer pair characteristics for qRT-PCR amplification

| Name | Orientaion | Sequence (5’-3’) | Final concentratin (nM) | Amplicn length | Sequence used in design process with position of amplicon and corresponding microarray oligo ID |
| --- | --- | --- | --- | --- | --- |
| VvAdh1 | Forward | AAG GTG ATC TTG GGT GAC TTT CA | 900 | 72 bp | AF194173 (1220-1291 bp)  Vv_10000593 |
|  | Reverse | CAA CCA GAC AGA TGC TCT CTT TCA | 900 |  |  |
| VvAgpL | Forward | gag tgt agt ttg gaa gac gat g | 300 | 111 bp | TC64860 (499-609 bp)  Vv_10001368 |
|  | Reverse | aca gga cag ata cca ctt gc | 300 |  |  |
| VvHP | Forward | agc aac aaa ttt tgg cag c | 300 | 88 bp | TC62606 (562-649 bp)  Vv_10001604 |
|  | Reverse | gcc att tgt cct ctt gct c | 300 |  |  |
| VvEtr | Forward | ttc gga agt tca gga gcc | 300 | 100 bp | TC52433 (606-705 bp)  Vv_10001788 |
|  | Reverse | cca ttc att cct atc tcc aag c | 300 |  |  |
| VvF3h | Forward | gca ggc tct atg gtt ttt tcc | 300 | 117 bp | TC53331 (347-463 bp)  Vv_10002511 |
|  | Reverse | gct tca tct tct cct cca cc | 300 |  |  |
| VvInv2 | Forward | cca acc aag gcg atc tat g | 900 | 76 bp | TC52500 (721-796 bp)  Vv_10004311 |
|  | Reverse | ttg agg cag tga tgc tgg | 900 |  |  |
|  | Probe^a^ | agc tca gct cta tgt ctt caa caa tgc tac | 100 |  |  |
| VvAcyt | Forward | cga gga ctt cca aac aca tc | 900 | 97 bp | TC57415 (2060-2156 bp) Vv_10003724 |
|  | Reverse | act ggg tca ttc ata gca tta c | 900 |  |  |
| VvLox | Forward | gcc atg agg aca aga aag atg | 900 | 116 bp | TC59834 (1227-1342 bp)  Vv_10003710 |
|  | Reverse | gtt gac ggc agc atg gtg | 900 |  |  |
| VvOlp | Forward | tcg cca gtc taa act act agg | 300 | 120 bp | TC63891 (759-878 bp)  Vv_10000872 |
|  | Reverse | cgt aga aaa gtt gtt gca tga g | 300 |  |  |
| VvSamt | Forward | tcc ttt acc ata agt agg ctg g | 900 | 128 bp | TC52353 (705-832 bp)  Vv_10000965 |
|  | Reverse | tcc gcc act gct ctc atc | 900 |  |  |
|  | Probe^a^ | acg gtg aat tct gcc cat cag atg ctc ata | 100 |  |  |
| VvSusy | Forward | TGT TAA GGC TCC TGG ATT TCA ATT A | 900 | 71 bp | TC38393 (2741-2817 bp)  Vv_10000177 |
|  | Reverse | AGC CAA ATC TTG GCA AGC A | 900 |  |  |
| VvWrky | Forward | tcc cat atg aga aag gaa gag g | 300 | 88 bp | TC66580 (944-1031 bp)  Vv_10004898 |
|  | Reverse | tcc gtc tac acc gca gtc | 300 |  |  |
| VvCaSy | Forward | TGG GAC GAC CGC ACA TC | 900 | 62 bp | TC78751 (1355-1416 bp) Vv_10001615 |
|  | Reverse | CAG CGC CAC AAG GTA AAA CAC | 900 |  |  |
|  | Probe^b^ | CCT CTG GCG TAA CCA C | 250 |  |  |
| VvCko | Forward | TGG GAC GAC CGC ACA TC | 900 | 60 bp | TC75658 (1222-1281bp) Vv_10009579 |
|  | Reverse | CAG CGC CAC AAG GTA AAA CAC | 900 |  |  |
|  | Probe^b^ | CCT CTG GCG TAA CCA C | 250 |  |  |
| VvGlc1 | Forward | TGCCATGTTTGACGAGGACAA | 900 | 75 bp | TC77213 (963-1037 bp) Vv_10000389 |
|  | Reverse | GGTTGTTTGTTAGGGAGGAAGAGC | 900 |  |  |
|  | Probe^b^ | ATGCTTCTCCAATTCTG | 250 |  |  |
| VvGlc2 | Forward | CCATCATCAGCTTCCTGGTCAAAA | 900 | 86 bp | TC76238 (618-703 bp) Vv_10002068 |
|  | Reverse | GTCCCGGGTGTTACCAATGTA | 900 |  |  |
|  | Probe^b^ | CCCCACTGCTTGTTAAC | 250 |  |  |
| VvGlc3 | Forward | CGCTTCTGGCGAATATATACCCTTA | 900 | 71 bp | TC92012 (624-694 bp) Vv_10010418 |
|  | Reverse | AGCGTAGGGAAGAGATATGTCCTT | 900 |  |  |
|  | Probe^b^ | ACTCAGGCAACCCC | 250 |  |  |

^a^TaqMan® Probe

^b^TaqMan® MGB Probe

**Table 2**. Performance characteristics of all qRT-PCR amplicons used in the study. The data for 18S/COX were calculated from the geometric mean of corresponding COX and 18S Ct values. Mean values of several individual measurements are represented with standard deviations. R^2^: correlation coefficient; E: efficiency of amplification.

| Amplicon | Linear regression | | | Ct range | Sample dilution factors used for quantification |
| --- | --- | --- | --- | --- | --- |
|  | Slope | R^2^ | E |  |  |
| VvAdh1 | -3.47±0.19 | 0.99±0.05 | 0.94±0.07 | 22.4 – 33.6 | 10^2^ – 10^3^  10 – 10^2b^ |
| VvAgpL | -3.65±0.21 | 0.99±0.07 | 0.88±0.07 | 24.2 – 31.9 | 10 – 10^2^ |
| VvHPt | -3.50±0.14 | 0.99±0.03 | 0.93±0.05 | 24.3 – 31.5 | 10 – 10^2^ |
| VvEtr | -3.38±0.57 | 0.97±0.08 | 1.02±0.22 | 27.4 – 35.6 | 10 – 10^2^ |
| VvF3h | -3.45±0.13 | 1.00±0.03 | 0.95±0.05 | 21.9 – 29.3 | 10 – 10^2^ |
| VvInv2 | -3.61±0.26 | 0.99±0.01 | 0.90±0.09 | 27.6 – 35.4 | 10 – 10^2^ |
| VvAcyt | -3.38±0.09 | 1.00±0.06 | 0.98±0.03 | 17.0 – 24.2 | 10^3^ – 10^4^ |
| VvLox | -3.62±0.07 | 1.00±0.03 | 0.89±0.02 | 22.9 – 30.5 | 10 – 10^2^ |
| VvOlp | -3.59±0.05 | 1.00±0.04 | 0.90±0.02 | 22.2 – 29.3 | 10 – 10^2^ |
| VvSamt | -3.72±0.36 | 0.99±0.00 | 0.90±0.08 | 27.4 – 35.9 | 10 – 10^2^ |
| VvSusy | -3.41±0.14 | 0.97±0.01 | 0.97±0.05 | 23.4 – 34.6 | 10^2^ – 10^3^  10 – 10^2b^ |
| VvWrky | -3.85±0.21 | 0.96±0.02 | 0.82±0.06 | 25.8 – 34.6 | 10 – 10^2^ |
| VvCaSy | -3.11±0.12 | 0.96±0.01 | 1.10±0.06 | 28.8 – 34.1 | 10 – 10^2^ |
| VvCko | -3.23±0.16 | 0.96±0.01 | 1.04±0.07 | 29.8 – 35.7 | 10 – 10^2^ |
| VvGlc1 | -3.48±0.02 | 1.00±0.00 | 0.94±0.01 | 26.5 – 34.9 | 10 – 10^2^ |
| VvGlc2 | -3.46±0.09 | 1.00±0.00 | 0.95±0.04 | 24.0 – 30.8 | 10 – 10^2^ |
| VvGlc3 | -3.44±0.29 | 0.96±0.00 | 0.96±0.11 | 28.3 – 34.9 | 10 – 10^2^ |
| COX | -3.45±0.10 | 1.00±0.01 | 0,95±0.02 | 19.5 – 26.50 | 10 – 10^2^ |
| 18S | -3.49±0.10 | 0.99±0.06 | 0.94±0.04 | 12.3 – 19.30 | 10^3^ – 10^4^  10^4^ – 10^5b^ |
| 18S/COX | -3.53±0.08 | 1.00±0.02 | 0.92±0.03 | 15.5 – 22.60 | / |

^b^ different dilution used in season 2005
